# Energy Absorption and Mechanical Performance of Functionally Graded Soft–Hard Lattice Structures

**DOI:** 10.3390/ma14061366

**Published:** 2021-03-11

**Authors:** Hafizur Rahman, Ebrahim Yarali, Ali Zolfagharian, Ahmad Serjouei, Mahdi Bodaghi

**Affiliations:** 1 Department of Engineering, School of Science and Technology, Nottingham Trent University, Nottingham NG11 8NS, UK; mohammed.rahman2018@my.ntu.ac.uk (H.R.); ahmad.serjouei@ntu.ac.uk (A.S.); 2 School of Mechanical Engineering, College of Engineering, University of Tehran, Tehran 11155-4563, Iran; ebrahim.me20@gmail.com; 3 School of Engineering, Deakin University, Geelong, VIC 3216, Australia; a.zolfagharian@deakin.edu.au

**Keywords:** energy absorption, bio-inspiration, graded cellular structures, finite element modeling, soft-hard composites, large deformations

## Abstract

Today, the rational combination of materials and design has enabled the development of bio-inspired lattice structures with unprecedented properties to mimic biological features. The present study aims to investigate the mechanical performance and energy absorption capacity of such sophisticated hybrid soft–hard structures with gradient lattices. The structures are designed based on the diversity of materials and graded size of the unit cells. By changing the unit cell size and arrangement, five different graded lattice structures with various relative densities made of soft and hard materials are numerically investigated. The simulations are implemented using ANSYS finite element modeling (FEM) (2020 R1, 2020, ANSYS Inc., Canonsburg, PA, USA) considering elastic-plastic and the hardening behavior of the materials and geometrical non-linearity. The numerical results are validated against experimental data on three-dimensional (3D)-printed lattices revealing the high accuracy of the FEM. Then, by combination of the dissimilar soft and hard polymeric materials in a homogenous hexagonal lattice structure, two dual-material mechanical lattice statures are designed, and their mechanical performance and energy absorption are studied. The results reveal that not only gradual changes in the unit cell size provide more energy absorption and improve mechanical performance, but also the rational combination of soft and hard materials make the lattice structure with the maximum energy absorption and stiffness, in comparison to those structures with a single material, interesting for multi-functional applications.

## 1. Introduction

Mechanical meta-materials demonstrate unprecedented mechanical properties that directly originate in their geometrical designs at different scales, in particular, small scales that are engineered to achieve unusual properties at the bulk (or macroscale) [1,2,3]. Some of these unique dimensionless properties are multi-stability [4,5], zero shear modulus [6], variable softening/hardening [7], snapping deformations [8] and a negative Poisson’s ratio (auxetics) [5,9]. It has been recently shown that the elastic stiffness and Poisson’s ratio of mechanical meta-materials can independently be tailored by rational design of not only the microarchitecture but also introducing bi-materials (i.e., hard and soft) [1]. Therefore, material properties of the solid constituent of meta-materials can also add a new level of design freedom in the design process of meta-materials. The most common approach for modeling meta-materials is properly considering the unit cell as a continuous solid medium with representative mechanical properties throughout the entire domain which comprises the overall bulk properties of the meta-materials [10,11].

Architected mechanical (multi) meta-materials, possessing a combination of several properties such as light weight, toughness, stiffness, damage tolerance and energy absorption have recently received the most attention for multifunctional applications in biomedicine, aerospace, automotive and civil engineering [12]. Mechanical energy absorption and structural integrity, similar to other phenomena, are coming by taking inspiration from nature. For instance, bone, antlers, woods, teeth, tusks and hooves are exemplars of energy absorption structure [13,14]. In general, conventional structures such as honeycomb, foam and sandwich panels, although good at energy absorption, are not optimally designed for that purpose. To address this issue and to enhance the energy absorption capacity of structures, we could take inspiration form nature and biological samples (e.g., plants and animals) [14]. Recently, architected mechanical (multi) meta-materials and bio-inspired cellular structures have been shown as good alternatives with high energy absorption for multi-functional applications [15]. In such architected structures, large strain at almost constant stress with high energy absorption can be tolerated without inducing high stress. Energy absorption principles in such advanced structures may be defined as the capacity to convert kinetic energy into other types of energy by means of elastic/plastic deformation, mechanical instabilities and failure [16].

Today, to produce advanced mechanical meta-materials, additive manufacturing (AM) technology (or three-dimensional (3D) printing) opens a new window in the production of such sophisticated structures. The most popular AM methods for fabricating lattice structures with geometrical complexity are powder-based (e.g., powder bed laser fusion (PBLF)) and filament-based (e.g., fused deposition modeling (FDM)) [17]. Moreover, many structures in the nature such as bone have distinctive properties. Therefore, lattice structures with graded changes in the size, properties and volume fraction, named functionally graded (FG) lattice structures, are exceptional alternatives [17,18,19]. Therefore, mechanical performance and energy absorption of two-dimensional (2D) and 3D lattice structures can be tuned by integrating graded size, multiple material (volume fraction of materials, soft-hard interactions and configurations) through rational design and triply periodic minimal surfaces (TPMS) or topology optimization [20,21,22,23,24,25,26,27].

From the design point of view, there are various architectures with different cell topology such as auxetic and honeycomb, which already are being used in the literature [1,15,28,29,30,31,32,33]. Among them, honeycomb cells are the most common topology used to study the quasi-static and dynamic behavior of such structures [34,35,36]. Corrugated and (meta-) sandwich structures are one of the common applications of lattice structures for the application of impact, bending resistance and energy absorbing systems [31,37,38,39,40]. The improvement of energy absorption of 2D lattice structures was experimentally and numerically investigated by gradual changes in the size of the unit cells [18,34]. Moreover, 3D mechanical lattice structures (made of TPMS) have been investigated recently for the application of energy absorbers [12,17,21,41,42]. For example, as a parametric study, the effect of unit cell size on the energy absorption capacity of Schwartz diamond graded porous structures made by TPMS was examined numerically and experimentally [21]. Sometimes, by taking inspiration from the shape memory effect of smart materials, a reversible energy absorption in meta-materials can be achieved by 4D printing technology [15].

Considering the open literature, there are a lot of studies on the investigation of mechanical performance and energy absorption of single material lattice structures and even with FG properties. However, multi-material mechanical lattice structures recently have increasingly gained attention as they provide more design freedom for tuning structural behavior. Therefore, the combination of different materials (soft and hard) and graded size could make promising light-weight structures with tunable and distinctive mechanical properties. In the present study, the mechanical performance (e.g., the quasi-static compression test) and energy absorption capacity of five types of graded cellular structures with soft and hard materials and two types of dual-material lattice structures are investigated. Mechanical lattice structures are made of thermoplastic co-polymer (TPC) as a soft material and polyamide (PA12) as a hard material with diversified mechanical properties. Five types of graded structures are rationally designed based on the gradual changes in the unit cells size and arrangement. Then, the mechanical performance and energy absorption capacity of each topologically designed structures are investigated by FEM for each material and verified by the experimental data on 3D printed lattice structures.

## 2. Materials and Methods

### 2.1. Structural Design

**Figure 1:**
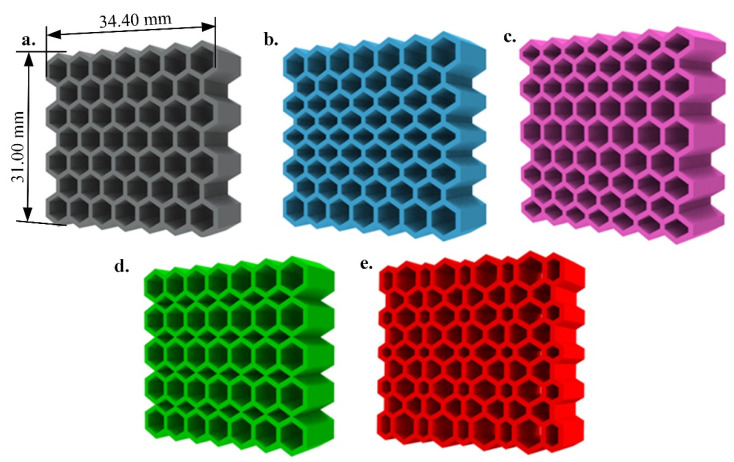
Geometrical drawing of the samples 1 to 5 (**a**–**e**).

To design the structures in the present paper, hexagonal cells with different unit cell length sizes and the same overall dimensions of $31.00\;\times \;34.40\;{\mathrm{mm}}^{2}$ are considered. Five types of graded size hexagonal lattice structures are designed with the same cuboid-like geometries and the same overall dimensions and thickness as shown in Figure 1. They are made of either soft (TPC) or hard (PA12) materials. Indeed, changing the unit cell size gradually means alerting the relative density of the structures. In sample 2 (Figure 1b), the unit cell length is gradually/linearly decreased from the top and bottom towards the center. In sample 3 (Figure 1c), the direction of changes is opposite of the sample 2. It means that the unit cell length is gradually/linearly increased from the perimeter of the structure to center. In sample 4 (Figure 1d), there is a discrete and unidirectional unit cell size in the structures. Finally, in sample 5 (Figure 1e), bidirectional unit cell size changes are introduced. For more details on experimental set-up and geometrical parameters of the samples, one may refer to [34].

**Figure 2:**
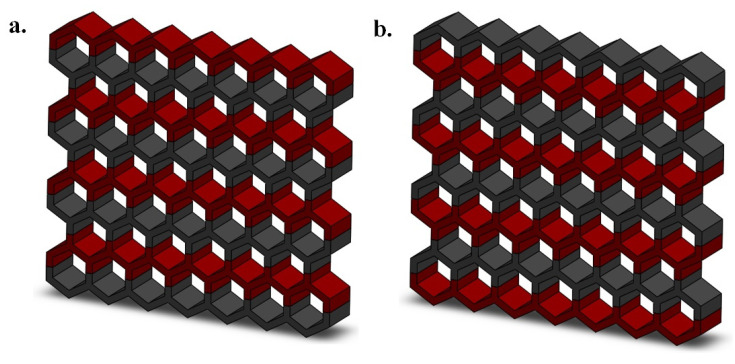
A schematic drawing of two types of dual-material architectured structures based on (**a**) polyamide–thermoplastic co-polymer (PA12–TPC) (sample 6) and (**b**) TPC-PA12 (sample 7). Red and grey colors indicate PA12 and TPC, respectively.

Furthermore, two dual-material lattices structures are designed by changing the distribution of materials (i.e., TPC and PV12) which are shown in Figure 2. In fact, by considering the regular honeycomb structure (sample 1), we design samples 6 and 7 with dual-material structures. As it can be seen in sample 6, half of the honeycomb unit cell (and the whole structure) is made of PA12 and the other side is made of TPC (Figure 2a). In sample 7, the opposite distribution of the materials is presented (Figure 2b).

### 2.2. Finite Element Modeling

3D finite element analysis is carried out using ANSYS Explicit Dynamics (2020 R1, 2020, ANSYS Inc., Canonsburg Pennsylvania, PA, USA). The explicit dynamics module used in the numerical software method controls the environment to ensure the simulations remain quasi-static, and the physics of the problem do not change. The criterion is through time scaling to speed-up the explicit solver; there is an energy summary which was checked. The kinetic energy is only a small portion of the internal energy suggesting that the inertia forces are small, and the simulation does in fact maintain a quasi-static scenario with the given explicit speed. One major advantage of this tool over static structural is the penetration mechanics of a higher structural penetration detection. In explicit dynamics, it is easier and more efficient to detect self-contact interaction within the energy absorbing lattice structure. Originally the static structural tool on ANSYS was used to carry out the quasi-static compression of all five samples. The simulations roughly required three hours each; however, one major problem that occurred was the inability of the solver to apply self-contacts on large deformations of the lattice, and this led to self-penetration of the structure being compressed eventually distorting the mesh greatly reducing accuracy and causing other solver errors. The improvement of shifting to the explicit dynamics tool resolves the mesh distortion and self-penetration problem where the lattice was able to be compressed and experience large deformations.

**Figure 3:**
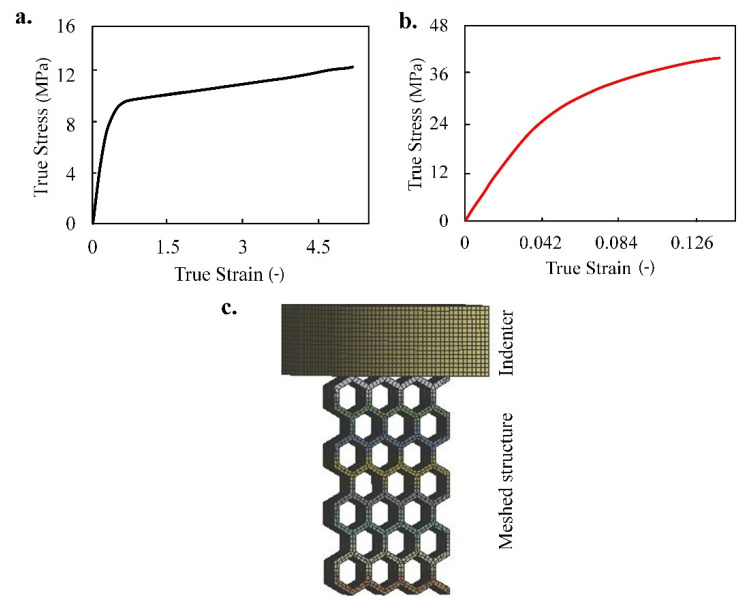
Uniaxial tensile test true stress—strain curve for performing numerical simulations for (**a**) TPC, (**b**) PA12 and (**c**) a sample of the meshed lattice structures (sample 1) and indenter.

To setup the quasi-static simulation, first the material data are imported using uniaxial test data of the both types of materials (i.e., TPC and PA12). To model TPC, a hyperelastic constitutive model, Mooney–Rivlin with two terms ($C_{10}$ and $C_{01}$), was used. The numerical software allows for curve fitting using experimental hyperelastic uniaxial test data to extract the Mooney–Rivlin material constants, $C_{10}$ and $C_{01}$ as 1.726 × 10^5^ Pa and 5.223 × 10^6^ Pa, respectively. The incompressibility parameter $D_{1}$ was automatically set to 0 (i.e., fully incompressible). For PA12, multi-linear isotropic hardening was used where the stress strain curve was implemented. J2 plasticity was also assumed. The numerical software has the capability to follow the assumed data for larger strain values when the curve was inputted. By having some experimental data to begin with, it can predict the continuous strain for a higher range. The true stress–strain behaviors of TPC and PA12 by Platek et al. [34] are presented in Figure 3. They used the MTS Criterion C45 universal strength machine to perform uniaxial tensile tests of 3D printed dumbbell samples based on TPC and PA12 [34]. For more details on experimental set-up, one may refer to [34]. TPC behaves like a hyperelastic material with high flexibility due to the elongation at break of 530%, while PA12 displays a more multilinear hardening material behavior with the elongation at break of 14%. From Figure 3, it can be concluded that the stiffness and elongation of TPC are much greater than those of PA12, with TPC enduring 530% elongation and PA12 enduring only 14%. Moreover, the density of TPC and PA12 were set to 1014 and 1010 $\mathrm{kg}/m^{3}$, respectively. The indenter that compresses the energy absorbing lattices should be set to have a rigid behavior in ANSYS in order to ensure the compressor does not experience deformation itself. Therefore, it is set to structural steel to ensure there is smooth compression with no deformation occurring at the indenter side. Noted that the die velocity (or strain rate) of the loading and coefficients of friction between the sample and top and bottom die plates were considered 1, 0.1 and 0.2 mm/s, respectively.

Symmetry was applied along the longitudinal direction (X direction) to achieve a faster solving time by reducing the number of elements within the mesh. However, an additional displacement condition was applied across the X direction to account for the central part moving along both the X and Y directions, which ensures the sample nodes are not restricted in the X-position and are free to move in Y. It was noted that there is no difference in using either a full model or a half model as they both result in the same deformation. To obtain the total force, further post processing in doubling the final force displacement graph to achieve the simulation of the lattice compression for the full model is required. The advantage this provided is a major reduction in the quantity of nodes and elements used which reduces the solver time to half the amount required to complete the 50% model. In addition, having the number of elements reduced allowing for improvement in the mesh quality and using a denser mesh such as one having 3–4 elements across the thickness of the wall. This ensures more accurate data are obtained across each element, and deformations are easier to detect at distinct points around the nodal locations of the lattice structure. Another possible idea that is considered during the simulation is using the mid-surface feature. Since there are quite a few thin solid beams on the lattice structure, the mid-surface allows conversion from solid to shell elements hence greatly reducing solver time and providing an improvement in detecting deflection across the lattice energy absorber. It should be mentioned that regarding the mesh type used in the present study, mesh type QUAD 4, solid linear elements with a medium span angle center and smoothing set to high were used (see Figure 3c, meshed hexagonal lattice structure, sample 1) and the indenter). Additionally, our simulations were verified against experimental data from Platek et al. [34]. In this respect, a convergence study was conducted to achieve the converged results accurately to two significant digits. The optimal mesh setup was used.

Additionally, a part transform function is applied in the model configurations. The benefit of applying a transform is that the indenter model is ensured to be in contact with the lattice structure pre-compression stage enabling a smoother transition to begin the quasi-static simulation by closing the initial contact gap. Therefore, the force-displacement results are obtained immediately. The body interaction function is the main advantage over static structural. This feature provides a higher self-penetration detection, ensuring that there is sliding contact within the lattice layers that compresses rather than distorting the lattice. In addition, to decrease the solving time of the simulations, mass scaling techniques by increasing density and stress wave density relationship are applied to the simulations. In fact, the mass scaling function reduces computational costs using the Courant–Friedrichs–Lewy (CFL) condition where the stability of a time step for explicit dynamics analysis is $\Delta t=scaling\;factor\;\times \;E_{min}/c$, where, $E_{min}$ is the size of the smallest mesh element, c is the speed of the stress wave and scaling factor is the numerical value for providing stability. From this, it is evident that the smallest mesh element on the lattice structure has the largest impact on reducing the solver run time. Enabling automatic mass scaling increases the mass of approximately 1% of the elements to increase the smallest time step and in turn reduces the number of total solver time steps. The mass scaling only occurs at 1% of the total mass for this case in order to ensure the original simulation conditions remain constant and in turn obtain more efficient results. Mass scaling should not occur at more than 5% of the elements as there may be unrealistic inertial effects. The option of stress wave density relationship is used to quickly solve pre-test simulations to ensure that the lattice is compressed correctly. Once it is verified that there are no compression problems, the density of the materials is changed back to normal to ensure the original problem is being simulated with the increased solver time.

## 3. Result and Discussion

**Figure 4:**
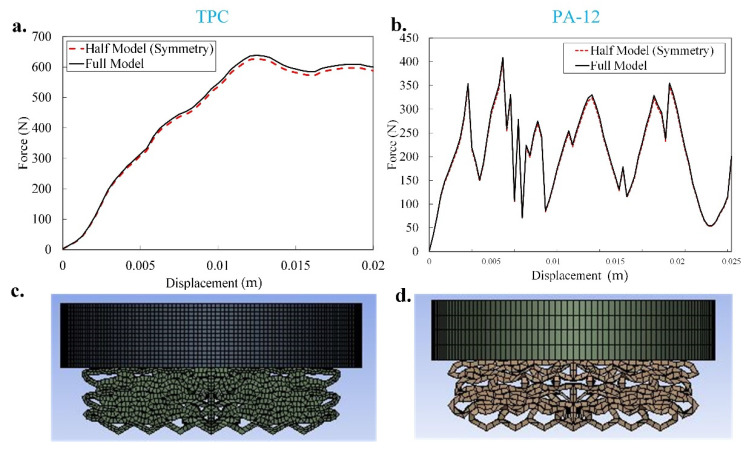
Numerical uniaxial force-displacement response for sample 1 based on (**a**) TPC and (**b**) PA12 in using both the full model and half model (symmetric). The deformed shape of the full model of (**c**) TPC and (**d**) PA12 at the end of loading.

In this section, all the results of mechanical compression test and energy absorption capacity of the five graded size lattice structures for single material (TPC and PA12) and dual-material mechanical lattice structures are presented. Since TPC and PA12 are very different from a material point of view, we aimed first to investigate the mechanical performance of the rational designed structures made by each material. Additionally, it is worthwhile to add both materials together in a dual-material structure for which the corresponding results are presented in Section 3.2. Notably, in the present simulations, an elasto-plastic model without considering the failure criteria was used, and the effects of shearing and cracking were ignored. Moreover, the finite element simulation took almost 6 h on an Intel(R) Core(TM) i5-7200U CPU @ 2.50 GHz (HP, Palo Alto, CA, USA) with 8 GB RAM. As mentioned in Section 2.2, based on the present problem, to reduce the simulations time, the symmetric boundary condition was considered. To make sure that there is no remarkable difference between full model and half model, uniaxial force-displacement response and their deformation mapping at the end of loading for sample 1 based on TPC and PA12 are presented in Figure 4. As shown in Figure 4, there is no significant difference between the results of full model and symmetric model. Therefore, all the simulations presented in the present paper were performed by considering the symmetric condition.

### 3.1. Graded Lattice Structures

**Figure 5:**
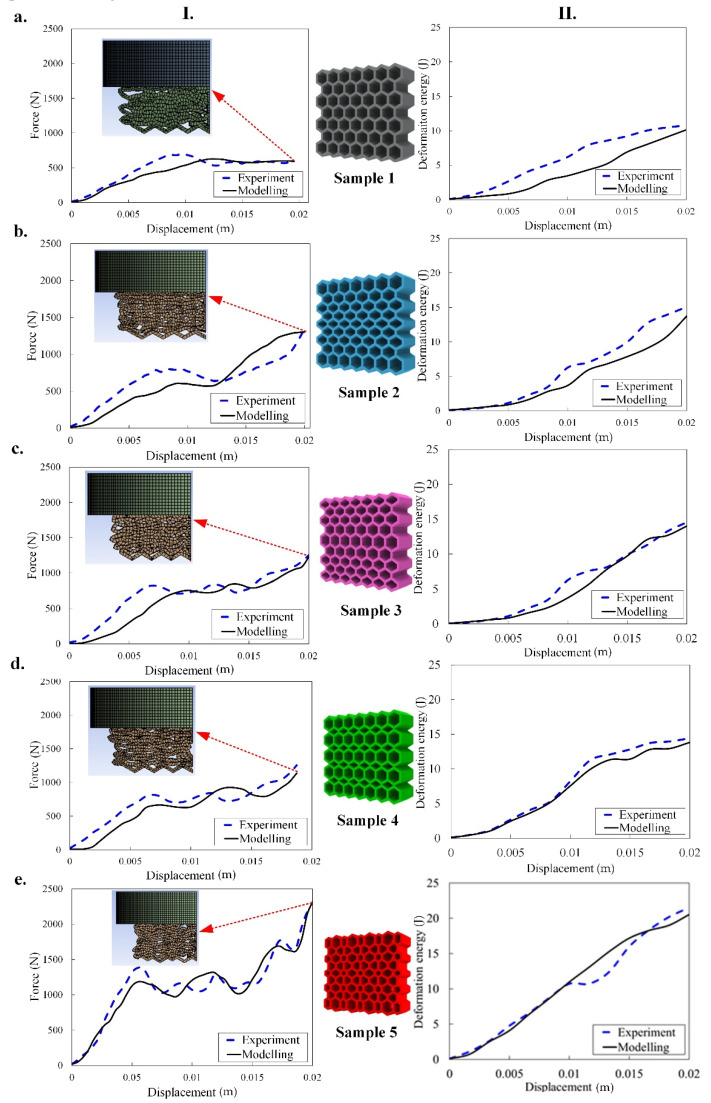
Experimental and numerical uniaxial force-displacement response (I) and deformation energy absorption (II) of the five samples (**a**–**e**) made of TPC under a quasi-static compression test.

**Figure 6:**
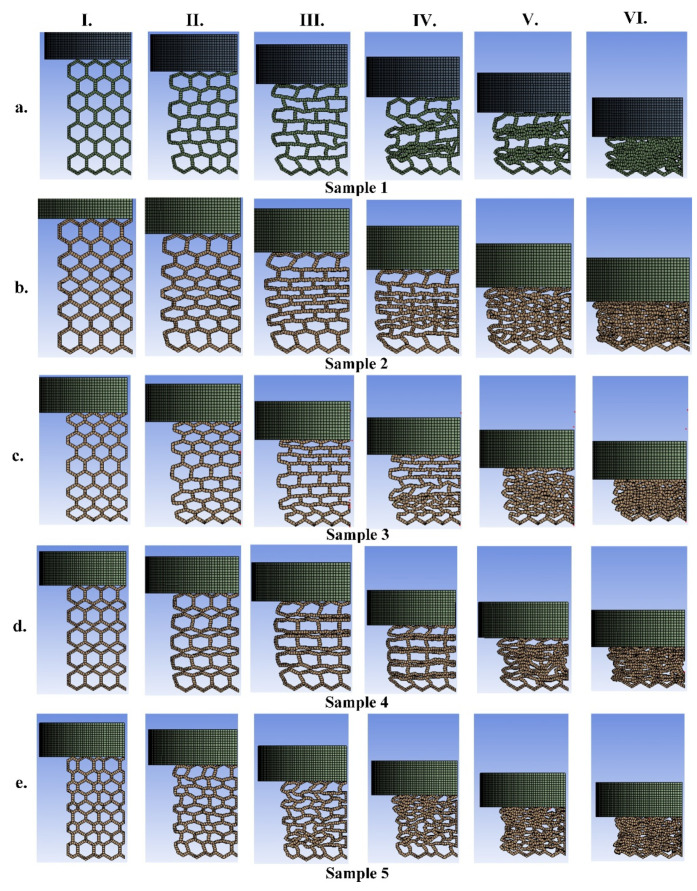
The deformation mapping of the samples 1 to 5 (**a**–**e**) made of TPC (each configuration from I to VI represents the current configuration at different displacements from 0.0033 to 0.02 m, with a displacement increment of 0.00334 m).

**Figure 7:**
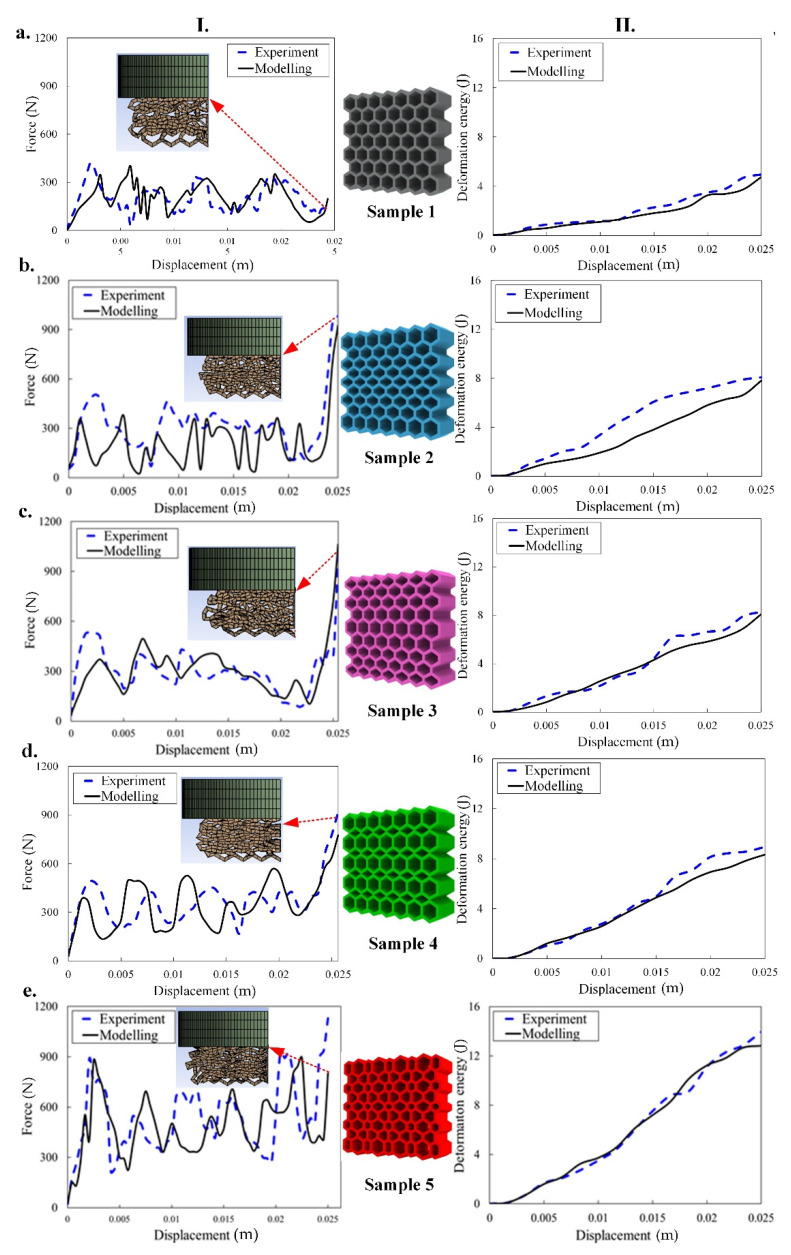
Experimental and numerical uniaxial force-displacement response (I) and deformation energy absorption (II) of the five samples (**a**–**e**) made of PA12 under a quasi-static compression test.

**Figure 8:**
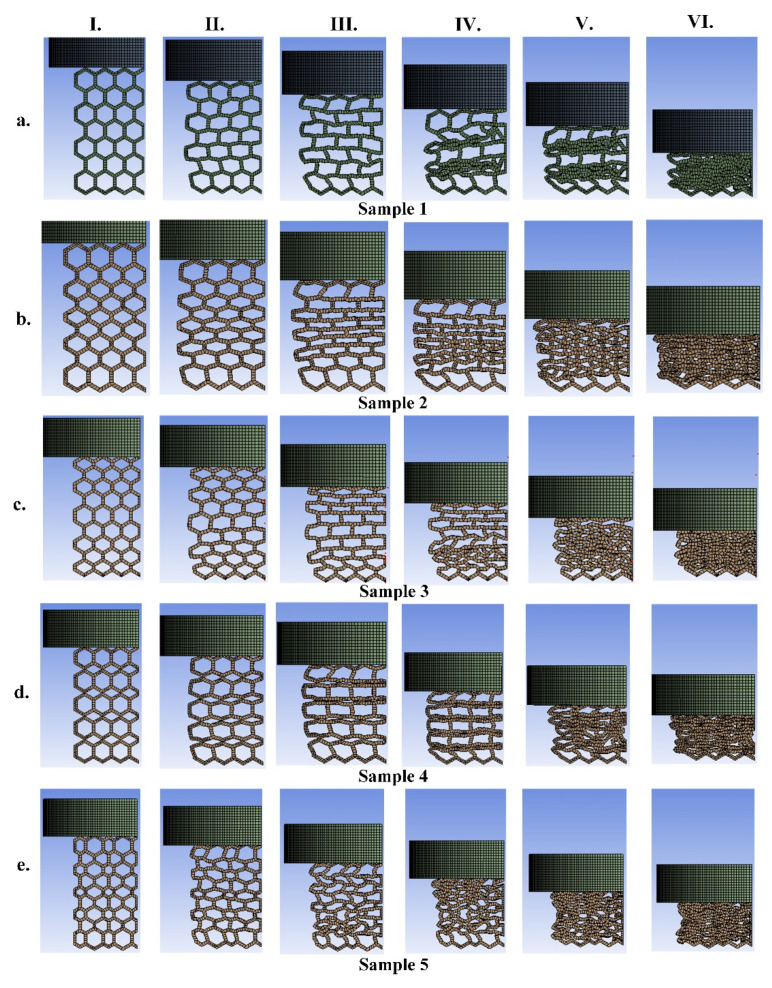
The deformation mapping of the samples 1 to 5 (**a**–**e**) made of PA12 (each configuration from I to VI represents the current configuration at different displacements from 0.0033 to 0.02 m, with displacement increment of 0.00334 m).

Here, the mechanical tensile test and energy absorption of the five types of graded size lattice structures for TPC and PA12 are separately presented. The numerical results including mechanical uniaxial force-displacement and energy absorption and their comparison with the experimental data of the TPC-based structures are shown in Figure 5. In addition, the deformation mapping of all five samples made of TPC over the increasing strain (from 0.0033 m to 0.02 m with displacement increment of 0.00334 m) is reported in Figure 6. Although experimental results in [34] did not include sample configurations, following other research works, we wanted to replicate the collapse from the top layers. Therefore, we considered different coefficients of friction between the sample and top and bottom die plates. As shown in Figure 5 and Figure 7, there is a good agreement between numerical results and experiments in terms of force-displacement graphs. At the end of the loading of TPC-based samples 1, 2, 3, and 5, we have the maximum agreement (with 3.5%, 3.2%, 2.1% and 3.2% errors, respectively) between experimental and numerical data for force-displacement. Additionally, for PA12-based samples 1, 2 and 3, we have the maximum agreement (with 4.0%, 3.1% and 2.6% errors, respectively) between experiments and numerical data for energy absorption at the end of loading. In such tests (compression), by increasing the strain up to a special value, the force increases sharply which means that the densification phenomenon occurred (see Figure 6 and Figure 8 (stages IV, V and VI), specially samples 2, 4 and 5). In other words, some coupling effects such as geometrical non-linearity, densification and plastic deformation induce an overall hardening during the loading. Additionally, one interesting result is that the energy absorption of graded size structures, especially sample 5 with bidirectional graded size unit cells, is higher than regular honeycomb structures without graded size, sample 1. Moreover, it is observed that in sample 5, due to the higher value of stiffness, the highest force and energy absorption are observed (see Figure 5e). In general, by comparing numerical results with the corresponding experimental results by Platek et al. [34], there is a good agreement between the present FEM results and experimental data under such a large deformation. Regarding the deformed configurations of samples (Figure 6 and Figure 8), deformations usually start from the top of the sample that is close to the indenter, and this observation was reported in most of experimental studies [15,17,18,21,43]. It should be mentioned that the difference between the experimental data of 3D printed structures and numerical simulations could be due to geometrical imperfection in the 3D printed samples and simplifications of the model used in the present study.

In the force-displacement plots (see Figure 5), there are some fluctuations which are caused by the initiation of the specimen collapse process (i.e., a local hardening behavior or local densification). In fact, due to geometrical non-linearity, the failure/break of the cell ligaments and the stiffness of the material under compression test (similar to buckling and bending loading), some collapses occur which suddenly result in softening behavior. Such softening–hardening behavior can be associated with the overall mechanical snap-though-like instability buckling of the structures. This phenomenon (i.e., collapse or local densification) can also be seen in Figure 6 and Figure 8. For example, in Figure 6a, we can see an irregular pattern transformation during the loading where collapse is initiated in the center of the sample. In sample 5, bidirectional functionally gradient, a regular collapse appeared simultaneously in both directions due to the dual-functionally gradient and eventually resulted in a smoother trend in the force-displacement plot (Figure 5e). In samples 2 and 3 due to the gradual decrease in the cell size of the samples from the margin to center and center to margin, respectively, the collapses started from center and margin, respectively. Additionally, from Figure 5d such collapse initiated from the unit cells with smaller size. Generally, it can be seen that when the ligaments are compressed and contact each other, the structures tend to harden at the end of the loading. In other words, the coupled effects of geometrical non-linearity, densification and plastic deformation cause overall hardening up to end of the loading. As a summary, it is concluded that collapse in TPC-based functionally graded structures initiated from cells with smaller sizes.

The numerical results comparison with the experimental data and their configuration during the loading of the PA12-based structures are shown in Figure 7 and Figure 8, respectively. Similar to TPC, it can be seen that the mechanical performance and energy absorption of the structures with graded size are enhanced in comparison to the sample with regular honeycomb cells (sample 1). Regarding Figure 5 and Figure 7, the value of the force (i.e., stiffness) of the TPC-based samples 2 and 3 and PA12–based samples 2–5 at the end of loading are almost similar. However, they behave differently during the loading. As mentioned before, applying bidirectional graded size in the unit cells, induces an increase in the geometrical stiffness of sample 5 and eventually results in higher value of force and mechanical energy absorption. In addition, in such complex non-linearity (both geometrical non-linear large deformation and material non-linearity), the FEM is successfully able to simulate the structures with an acceptable agreement in comparison with experimental data. In this respect, it is seen that most of the experimental features are predicted by the FEM. The trends of the pattern transformation of the PA12-based structures are similar to those structures based on TPC, except sample 1 (i.e., Figure 8a). The collapse initiation from center in Figure 8a (i.e., PA12-based hexagonal structure) is more linear than that in Figure 6a (i.e., the TPC-based hexagonal structure). Overall, from the force-displacement plots perspective we see more fluctuation in PA12-based structures in comparison to those structures based on TPC. This is due to the higher stiffness of the PA12. From the pattern transformation perspective, however, a similar pattern is almost seen.

### 3.2. Dual-Material Lattice Structures

**Figure 9:**
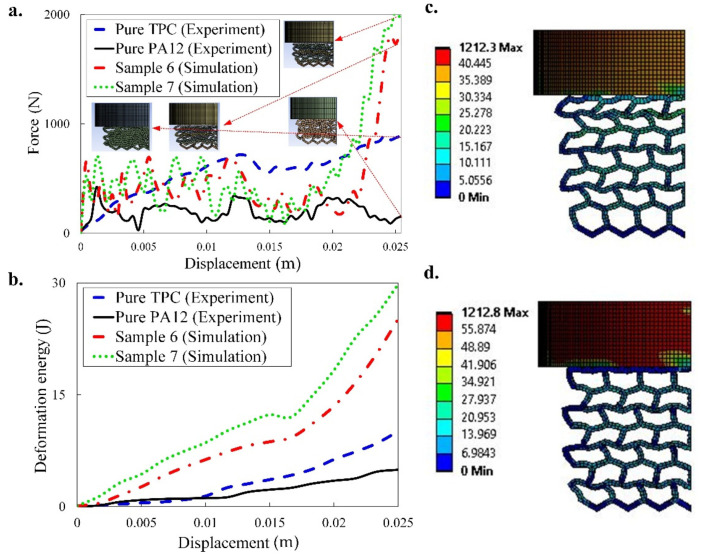
Numerical uniaxial force-displacement response (**a**), deformation energy absorption (**b**) and von Mises stress contour (in MPa) at state III (**c**,**d**) of the PA12-TPC (sample 6) and TPC-PA12 (sample 7), respectively, in comparison with the experimental results of pure TPC and PA12 under quasi-static compression test (see Figure 2 for the difference between the two samples).

**Figure 10:**
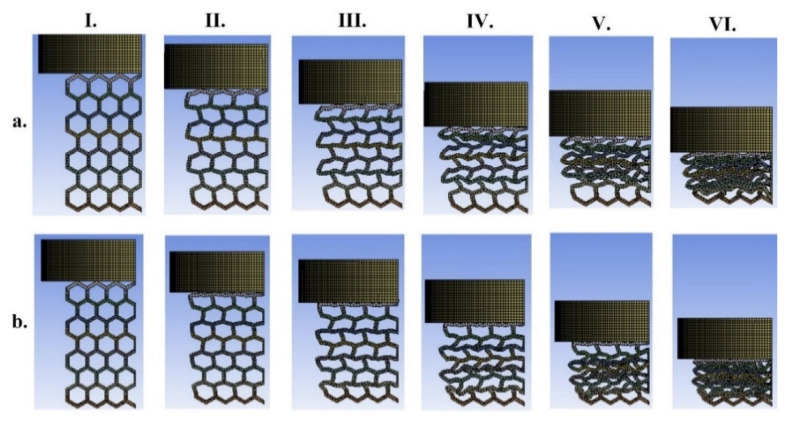
The current configuration of the dual-material mechanical lattice structures during loading (I to VI) for (**a**) PA12-TPC (sample 6) and (**b**) TPC-PA12 (sample 7) (see Figure 2 for the difference between the two samples). Note that applied displacement in I to VI are increased from 0.0033 to 0.02 m, with a displacement increment of 0.00334 m.

In this part, the mechanical compression test and energy absorption of the two types of dual-material mechanical lattice structures based on the combination of TPC and PA12 are presented (samples 6 and 7 in Figure 2). The force-displacement and energy absorption of sample 6 and 7 and the configuration of them during the loading are shown in Figure 9 and Figure 10, respectively. The experimental data for pure TPC and PA-12 (sample 1 in Figure 1) are also added for reference. From the pattern transformation point of view (Figure 10), it can be concluded that unlike single material hexagonal lattice structure (Figure 6a and Figure 8a), the dual-material hexagonal structure has a linear pattern (shape) transformation during the loading. This is due to the distribution of hard and soft materials with high and low stiffness in dual-material hexagonal structure. Meanwhile, two samples 6 and 7 have almost the same configuration during the loading (see Figure 10a,b), and extensive rotations and bending of ligaments around the connecting nodes occur throughout the deformation. It can be seen from Figure 9a that for both samples 6 and 7, initial linear elastic deformation occurs up to very small displacement of around 0.0005 m, after which plasticity starts through steady ductile-like plateau collapse region with load oscillations. The oscillations in the plateau region of experimental data are attributed to the collapse of layers or ligaments which have porosity and defects due to manufacturing process. At the end of the force plateau after the successive contact of all the ligaments, densification, where a sharp increase in force occurs, starts at 0.0022 and 0.00175 m for samples 6 and 7, respectively. It is worth mentioning that although the stiffness of PA12 is much larger than TPC, the force-displacements graphs presented in Figure 9 show a contradictory trend in the plateau and densification regimes. At small displacement regimes up to 0.0025 m, the PA12 shows larger force (see Figure 9a) and as displacement increases, the average and maximum force for the pure PA12 remains lower than pure TPC. It seems that pure PA12 goes to plastic regime earlier that TPC and therefore its load bearing and energy absorbing capacity is less than that of the pure TPC sample. As mentioned previously, deformations usually start from the top of the sample that is close to the indenter, and this observation was reported in most of experimental studies [15,17,18,21,43]. Although we do not have any experimental results on multi-material samples, we wanted to have the collapse from the top layers. Therefore, we considered different coefficients of friction between the sample and top and bottom die plates. Therefore, force-displacement response for samples 6 and 7 are slightly different due to asymmetric conditions in the top and bottom surfaces. Moreover, based on the Von–Mises stress contour plotted in Figure 9c,d, despite their deformed shapes, there are also differences between stress flow and the magnitude of the von Mises stress in the ligaments of the sample 6 and 7.

The numerical results presented in Figure 9 generally show that by rational combination of materials (with extremely different properties) and architectural design we are able to achieve the best structures suitable for energy absorption applications. By comparing the energy absorption and force-displacement of the dual-material honeycomb structure with those pure TPC or PA12 structures, the values of such parameters are four times higher than the corresponding parameters in single materials. As a result, both motifs functionally graded and multi-material can raise the mechanical performance and energy absorption capability of lattice structures.

## 4. Conclusions

In the present paper, the mechanical performance and deformation energy absorption of homogeneous and graded size lattice structures made of single and dual-materials TPC and PA12 were studied. Five different graded size hexagonal lattice structures made from TPC and PA12 were designed, and their mechanical compressive force-displacement and energy absorption were investigated. Then, by combination of the two materials TPC and PA12, two different hexagonal structures were designed and compared for their energy absorption. The presented numerical simulations were performed using FEM implemented in ANSYS by considering elastic/plastic and hardening material behaviors. It was found that the structures with gradual change in their topologies have a higher energy absorption and mechanical performance in comparison with the corresponding regular hexagonal lattice structure. In addition, dual-material mechanical lattice structures (i.e., samples 6 and 7) have higher energy absorption compared to their corresponding single material. Meanwhile, the present numerical results for such complex structures and non-linearity have a good agreement with the corresponding experimental data. Moreover, the coupled phenomena including geometrical non-linearity, densification and plastic deformation cause overall hardening up to the end of the loading. In conclusion, although (functionally) graded size lattice structures have a higher mechanical performance in comparison with regular lattice structures, functionally graded soft-hard lattice structures give the highest potential for energy absorption applications.

## Data Availability

Data sharing is not applicable to this article.
